# Childhood nocturnal enuresis—a marker for pelvic floor disorders and urinary tract symptoms in women?

**DOI:** 10.1007/s00192-020-04345-x

**Published:** 2020-05-30

**Authors:** Jwan Al-Mukhtar Othman, Sigvard Åkervall, Mattias Molin, Maria Gyhagen

**Affiliations:** 1 Gothenburg Continence Research Centre, Institute of Clinical Sciences, Sahlgrenska Academy at Gothenburg University, Gothenburg, Sweden; 2 Department of Obstetrics and Gynecology, Sahlgrenska University Hospital, 416 85 Göteborg, Sweden; 3 Statistical Consultancy Group, Gothenburg, Sweden; 4 Department of Obstetrics and Gynecology, Södra Älvsborgs Hospital, Borås, Sweden

**Keywords:** Childhood nocturnal enuresis, Lower urinary tract symptoms, Nulliparous women, Pelvic floor disorders, Pelvic organ prolapse, Fecal incontinence

## Abstract

**Introduction and hypothesis:**

A systematic survey on the association between childhood nocturnal enuresis (CNE) and adult pelvic floor disorders (PFDs) has not been presented previously. The aim was to describe the prevalence of PFDs and lower urinary tract symptoms in nulliparous women, with or without a history of CNE, at the age of ≥ 5 years.

**Methods:**

This national survey of urinary (UI) and fecal incontinence (FI) and symptoms of pelvic organ prolapse (sPOP) was a random sample of 20,000 nulliparous women aged 25–64 years conducted in 2014. Women ≥ 5 years of age having CNE were compared with those without the condition. Fisher’s exact test and logistic regression adjusted for BMI and age were used to analyze differences between groups.

**Results:**

The response rate was 52% and 10.2% of adult women reporting CNE. One or more PFDs occurred in 38.3% of women with CNE compared to 23.8% in those without CNE (*p* < 0.0001). Mixed UI had the strongest association with CNE, odds ratio (OR) 2.63 (95% CI 2.03–3.40). The rate of FI was 11.2% in the non-CNE group and 16.8% in those with CNE (*p* < 0.0001) and sPOP 2.6% in the non-CNE and 4.8% in the CNE group (*p* = 0.0004), respectively. The prevalence of lower urinary tract symptoms was consistently higher in women with a history of CNE: overactive bladder 32.6% versus 18.4% (OR 2.34 95% CI 2.03–3.40), daytime micturition ≥ 8/day 29.6% versus 24.0% (*p* < 0.0001), and nocturia ≥ 2/night 12.4% versus 7.8% (*p* < 0.0001) in the CNE group.

**Conclusion:**

PFDs and lower urinary tract symptoms in nulliparous women were approximately doubled in women with a history of CNE and could therefore act as a strong confounding factor.

**Electronic supplementary material:**

The online version of this article (10.1007/s00192-020-04345-x) contains supplementary material, which is available to authorized users.

## Introduction

Childhood nocturnal enuresis (CNE) implies both a symptom and a condition of intermittent incontinence that occurs during periods of sleep in children ≥ 5 years of age and occurs in 7–10% of children at the age of 7 years [1]. Most reports have shown that CNE is more common in boys (2:1), but this gender difference diminishes with age [2]. In all except 0.5–1.7% of those with CNE, the condition will resolve spontaneously up to the age of 16–17 years [2]. In adult women nocturnal enuresis (NE) increases minimally with age from 0.3% at 20 years to 1.2% at about 65 years of age [3]. If CNE is still present at the age of 7 years, the child has a 5–10% risk of having persistent symptoms in adulthood [4]. Many epidemiological reports have shown a strong and consistent familial aggregation of CNE [2]. Clinically, it is a heterogeneous condition with a complex mode of inheritance [5]. Twin studies have shown that CNE probably has a genetic background, although a third of cases have been shown to be sporadic and environmental factors are thought to exercise a strong modulating effect [2]. The pathophysiology of CNE is thought to entail a dysfunction in the central nervous system regulation of sleep, leading to a greater depth of sleep and impaired arousal to the sensation of a full bladder [2].

At present there are only a few studies, using groups of women with mixed parity, that have looked into the association between CNE and selected lower urinary tract symptoms in adult women [6–12]. Since factors related to childbirth are strong predictors for pelvic floor disorders (PFDs) later in life, parous women should be excluded in such studies to avoid the confounding influence of childbirth. To date, to our knowledge no such study has been performed, nor has there been a study describing the effect of CNE on a wider spectrum of PFDs in adult women. The aim of this study was therefore to investigate the association between a history of CNE at ≥ 5 years of age and a wide spectrum of PFDs in a large, nationwide, randomly selected cohort of adult nulliparous women aged 25 to 64 years.

## Materials and methods

This prospective cohort study used data from a national population register linked to information from a questionnaire survey about current symptoms of PFDs. The postal and internet-based questionnaire survey was conducted in 2014. Ethical approval for the study was obtained from the Regional Ethical Review Board in Gothenburg (reference no. 776–13; November 18, 2013), and all women received written information and gave their consent to participate by answering the questionnaire.

The potential study population was identified by Statistics Sweden from the Total Population Register and comprised women registered in Sweden (> 99%) who had not given birth and were 25–64 years of age. The lower limit of the age span was set to 25 years because studies of adolescent girls and young women indicate that the prevalence of UI seems to stabilize at a minimum during the 3rd decade [13]. The upper limit was set to 65 years because confounding, age-dependent comorbidities potentially influence the prevalence of PFDs, which is known to increase rapidly from the 7th decade.

A total of 20,000 of the 625,810 eligible nulliparous women resident in Sweden were randomly invited to participate. The 20,000 participants comprised four independent, random samples, stratified by decades of age (25–34 to 55–64 years). A letter about the study, which included log-in credentials to an internet site, was sent to all women, requesting them to give their written informed consent. The introductory letter was followed by postal questionnaires. The questionnaire (internet and postal versions) was returned by 10,187 women after 3 mailing cycles during a 4-month period. Internet was used by 52% of the women. The answers revealed that 194 women were pregnant, 525 were parous, and a further 264 declined participation or returned an unusable form. These women were excluded, plus another seven because of missing information about parity. Misdiagnosis of parity was related to immigration (337/525). The final study population thus comprised 9197 women. A flow diagram for the selection of the study population has been presented earlier [14]. This study is the fourth report of the Swedish Pregnancy Obesity and Pelvic Floor project in nulliparous women. Earlier studies addressed the age-dependent prevalence of UI, sPOP, and overactive bladder [14–16]. The questionnaire included questions about current height and weight, childhood NE, urinary incontinence (UI) or fecal incontinence (FI), genital prolapse, nocturia, urgency, and menstrual status (information about current pregnancy, hysterectomy, menopausal status, and hormone treatment). Three separate validated questionnaires created by Sandvik et al., Tegerstedt et al., and Jorge and Wexner [17–19] were combined into one questionnaire, which is available in an earlier report [14].

CNE was defined according to the International Children’s Continence Society (ICCS) as both a symptom and a condition of intermittent incontinence that occurs during periods of sleep at the age ≥ 5 years [1] and regardless of the absence or presence of lower urinary tract symptoms. The women answered the question “Were you a bedwetter during childhood?” followed by “If yes, at what age did it stop?” UI, subtypes of UI, and nocturia were defined according to the International Urogynecological Association (IUGA)/ International Continence Society (ICS) definition [20]. UI was defined by the question “Do you have involuntary loss of urine?” Participants reporting UI were also asked if the incontinence had been present for > 10 years (UI > 10 years). Stress UI (SUI) was defined as involuntary loss of urine in connection with coughing, sneezing, laughing, or lifting heavy items; urge UI (UUI) was present if loss of urine was in connection with a sudden and strong urge to void and mixed UI (MUI) if both components were present. In this study a more restricted definition of nocturia (≥ 2 times/night) was used. Overactive bladder (OAB) was defined according to the IUGA/ICS definition as urgency with or without incontinence, usually accompanied by frequency and nocturia [20]. Frequency of leakage was stratified into four categories: “less than once a month,” “once or more per month,” “once or more per week,” and “every day and/or night.” The amount of leakage was categorized into “a few drops” vs. “small amounts” and “large amounts.” The mental impact perceived by incontinent women was dichotomized into “not bothersome” (no problem/a small nuisance) and “bothersome UI” (some bother/much bother/a major problem).

Symptoms of pelvic organ prolapse (sPOP) were defined by the question “Do you have a sensation of tissue protrusion (a vaginal bulge) from your vagina?," with the alternative answers never/infrequently = no, sometimes/often = yes [21]. Fecal incontinence (FI) was defined by affirming involuntary loss of liquid or solid feces (infrequently/sometimes/often) [19]. Body mass index (BMI) (kg/m^2^) was calculated from weight and height given in the questionnaire. Characteristics of responders and non-responders have been described in detail earlier [14].

## Statistical analysis

Descriptive data for continuous variables were presented as mean and standard deviation (SD) and categorical data as number and percentage. The prevalence of different aspects of PFDs was calculated for all women. In each analysis missing data were accounted for and excluded. Comparison between groups was analyzed with Fisher’s exact test for categorical variables and Student’s t test for continuous variables. Results were presented as number, percentage, 95% CI, and *p* values. No adjustment was made for multiple testing. The difference between groups was also analyzed using a logistic regression model, taking age and BMI into account. Results were presented as adjusted odds ratio (OR) and 95% CI. *p* < 0.05 was considered statistically significant. The trend between independent age groups was analyzed with Mantel-Haenszel statistics. Statistical analyses were performed using SAS 9.4 (SAS Inc., Cary, NC, USA).

## Results

The overall response rate was 52.2%, 44.7% among the youngest (25–34 years), 47.4% (35–44 years), 54.8% (45–54 years), and 62.4% among the oldest (55–64 years) women. The number of eligible women was 9197 of which 98.6% answered the question about CNE (9066). The 926 women with CNE (10.2%) were younger (2.1 years), less often post-menopausal (10.9% versus 17.9%, *p* < 0.0001), and had more often a family history (mother) of UI compared with the 8140 women without CNE (Table 1). CNE was more prevalent in younger women aged 25–34 years (11.4%) compared to the older women aged 55 to 64 years (7.3%), *p* for trend < 0.0001, as shown in Supplementary Materials, Table S1.

**Table 1:** Cohort characteristics according to the presence of childhood nocturnal enuresis

Cohort characteristics	CNE *N* = 926		No CNE *N* = 8140		*p* value*
	Mean (SD)		Mean (SD)		
Age (years)	38.9 (11.1)		41.0 (11.9)		< 0.0001
Weight (kg)	70.1 (16.2)		69.2 (15.2)		= 0.11
Height (cm)	167.0 (7.0)		166.9 (6.7)		= 0.62
BMI (kg/m^2^)	25.1 (5.5)		24.8 (5.2)		= 0.18
	Proportions of categorical variables				
	n	% (95%CI)	n	% (95%CI)	
BMI ≥ 30 (kg/m^2^)	139	15.2 (12.9–17.7)	1113	13.9 (13.2–14.7)	= 0.29
Medication for OAB	5	0.6 (0.2–1.3)	67	0.8 (0.6–1.1)	= 0.44
Medication for UI	5	0.5 (0.2–1.3)	25	0.3 (0.2–0.5)	= 0.23
Surgery for UI	7	0.8 (0.3–1.6)	21	0.3 (0.2–0.4)	= 0.02
Estrogen treatment	19	2.1 (1.3–3.3)	161	2.0 (1.7–2.3)	= 0.90
Postmenopausal	101	10.9 (9.0–13.0)	1458	17.9 (17.1–18.8)	< 0.0001
Hysterectomy	34	3.7 (2.6–5.2)	318	3.9 (3.5–4.4)	= 0.79
Mother had UI	194	47.2 (42.3–52.2)	1154	30.4 (28.9–31.9)	< 0.0001

Note: *CNE* denotes childhood nocturnal enuresis; *BMI* denotes body mass index; *OAB* denotes overactive bladder; *UI* denotes urinary incontinence

*For comparison between the two groups, Fisher’s exact test was used for dichotomous variables and Student’s t test for continuous variables

^**¶**^For the question of family history of UI (mother), 4859 of 9066 women answered “Do not know” (53.6%) and were excluded from the analysis

However, age had a weak effect on the association between adult PFDs and CNE, as shown in Supplementary Material, Table S2. The rate of missing data varied between 0.3% and 1.9% for different outcomes.

**Fig. 1:**
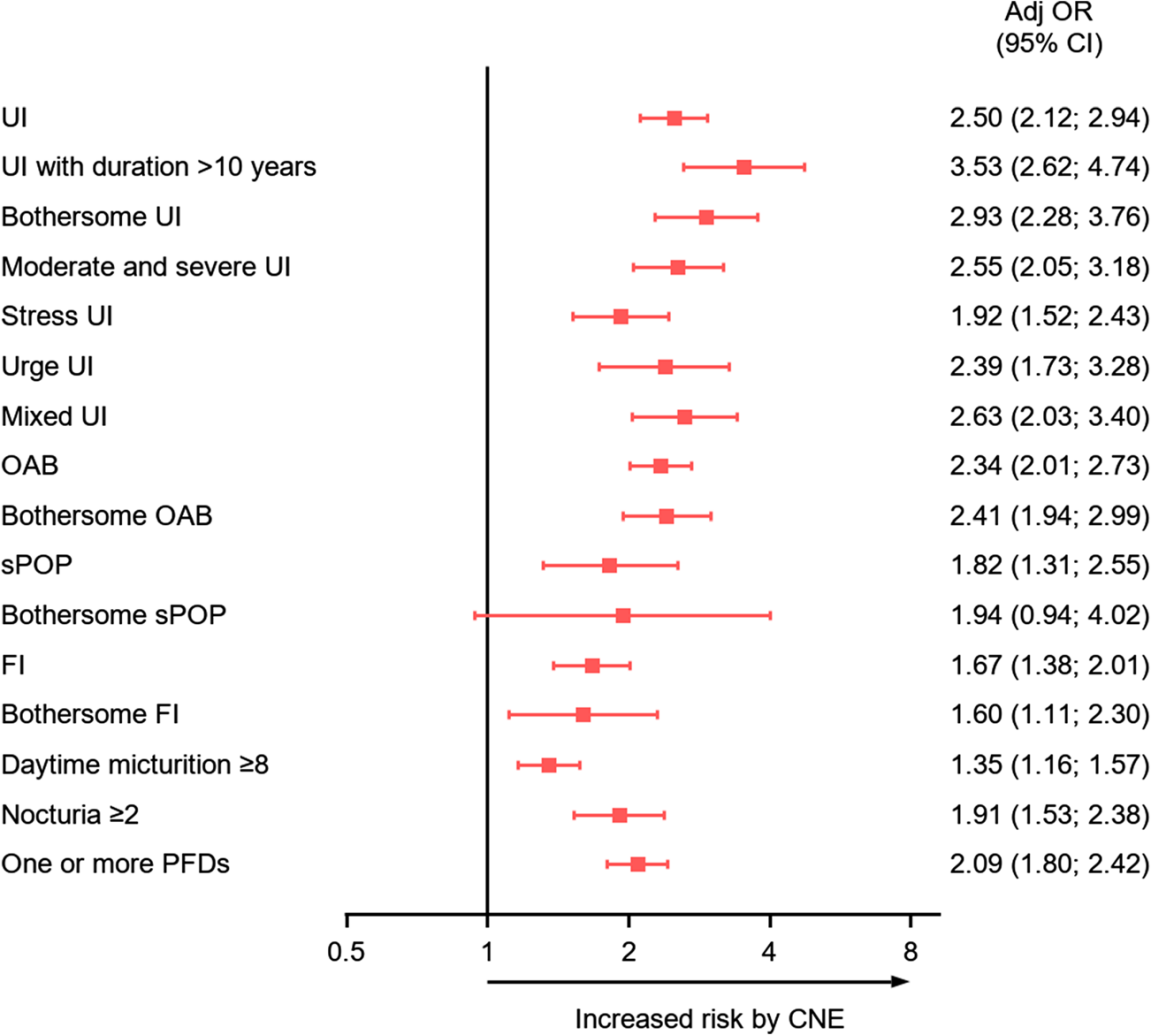
Risk for pelvic floor disorders in nulliparous women with a history of childhood nocturnal enuresis. UI denotes urinary incontinence; moderate and severe UI was defined according to Sandvik’s severity index.^15^ SUI denotes stress UI; UUI denotes urge UI; MUI denotes mixed UI; OAB denotes overactive bladder; sPOP denotes symptoms of pelvic organ prolapse; FI denotes fecal incontinence; PFD denotes pelvic floor disorder. CNE denotes childhood nocturnal enuresis. *Odds ratio and 95% confidence limits were calculated using logistic regression analysis with adjustment for age and BMI (kg/m^2^)

The prevalence of UI was doubled in women with CNE (14.8% compared to 28.6%, OR 2.50; 95% CI, 2.12–2.94; *p* < 0.0001), and the prevalence of all subtypes of UI was also about doubled in the CNE-positive women (Table 2). The strongest association was found for mixed UI (4.2% to 9.3%, OR 2.63; 2.03–3.40; *p* < 0.0001). In women without CNE, the prevalence of OAB was 18.4% compared with 32.6% in women with CNE (OR 2.34; 95% CI 2.03–3.40, *p* < 0.0001). Daytime micturition ≥ 8 was 24.0% compared with 29.6% in the CNE group (*p* < 0.0001) and for nocturia ≥ 2/night 7.8% compared with 12.4% (*p* < 0.0001). Other PFDs, not related to bladder symptoms, were also consistently higher in women with CNE. FI was 11.2% in the non-CNE group and 16.8% in the CNE group (*p* < 0.0001) and sPOP 2.6% compared with 4.8% (OR 1.82; 95% CI 1.31–2.55; *p* = 0.0004) (Fig. 1, Table 2*).*

**Table 2:** Prevalence of pelvic floor disorders and lower urinary tract symptoms

Aspects of pelvic floor disorders	CNE *N* = 926		No CNE *N *= 8140		*p* value ^**ƒ**^
	n	%	n	%	
Urinary incontinence	261	28.6	1195	14.8	< 0.0001
Bothersome UI	93	10.0	317	3.9	< 0.0001
Duration of UI > 10 years	67	7.2	172	2.1	< 0.0001
Moderate and severe UI^**§**^	126	13.6	528	6.5	< 0.0001
UUI	50	5.4	204	2.5	< 0.0001
SUI	98	10.6	480	5.9	< 0.0001
MUI	86	9.3	340	4.2	< 0.0001
sPOP	44	4.8	213	2.6	= 0.0004
Bothersome sPOP	9	1.0	41	0.5	= 0.074
FI	155	16.8	904	11.2	< 0.0001
Bothersome FI	11	1.3	55	0.7	= 0.048
OAB	300	32.6	1486	18.4	< 0.0001
Bothersome OAB	119	13.1	520	6.5	< 0.0001
Daytime micturition ≥ 8	273	29.6	1932	24.0	< 0.0001
Nocturia ≥ 2	114	12.4	629	7.8	< 0.0001
≥ 1 PFDs	355	38.3	1940	23.8	< 0.0001

Note: *CNE* denotes childhood nocturnal enuresis; *UI* denotes urinary incontinence; *UUI* denotes urge urinary incontinence; *SUI* denotes stress urinary incontinence; *MUI* denotes mixed urinary incontinence; *sPOP* denotes symptoms of pelvic organ prolapse; *FI* denotes fecal incontinence; *OAB* denotes overactive bladder; *PFD* denotes pelvic floor disorder (UI/sPOP/FI)

^**§**^Moderate and severe UI are considered to be more severe forms of urinary incontinence

^**ƒ**^For comparison between the two groups Fisher’s exact test was used for dichotomous variables

Surgery for UI was rare in both groups (< 1%), but significantly more prevalent in the CNE-positive group (0.3 versus 0.8%, *p* = 0.02), whereas medication for UI and OAB was similar.

## Discussion

The main finding of this study was that the prevalence of a wide spectrum of pelvic floor disorders and lower urinary tract symptoms was approximately doubled in nulliparous women with a history of childhood NE. The prevalence of a micturition frequency ≥ 8/day, nocturia ≥ 2/night, OAB, sPOP, FI and ≥ 1 PFDs was higher in nulliparous women with a history of childhood NE compared to women without a history of childhood NE suggesting that there may be at least one common causal factor linking childhood NE to these adult pelvic floor disorders.

The earliest studies about a possible association between CNE and PFDs in adulthood demonstrated an association with adult UUI but not SUI [6–8]. These observations were later confirmed for UUI (OR 2.7, *p* < 0.005) and severe UI (OR 2.9, *p* = 0.002) in the study of Kuh et al. in 1333 predominantly multiparous women aged 48 years [9]. In a clinical evaluation of 1021 women on annual gynecological examination, Gurbuz et al. found an association between CNE and SUI and FI but no association with MUI and UUI [10]. Fitzgerald et al. showed an association with adult UUI weekly or more often (OR 2.7), but no association with urinary frequency ≥ 8/day, nocturia ≥2/night, urgency, or SUI weekly and more often [11]. D’Ancona et al. interviewed patients presenting with UI at an outpatient clinic (585 women, 76 men, mean age 54 years) and found that those with MUI and UUI in adult life were more likely to have a history of CNE than those with adult SUI [12]. Thus, overall, these studies, although they preferentially focused on UI, were conflicting and were based on smaller, often convenience samples of women with mixed parity.

The most important difference between these earlier studies and the present study was that the major confounding factor on the incidence of PFDs caused by childbirth was excluded by using only nulliparous women. The current study was also based on a large, nationwide, and randomly selected cohort. With this design, it was shown that CNE was associated with a wide range of lower urinary tract symptoms: UI, SUI, UUI, MUI, moderate to severe UI, UI > 10 years, urinary frequency ≥ 8/day, nocturia ≥ 2/night, and urgency/OAB. More importantly, CNE was also associated with sPOP and FI.

## Strengths and weaknesses

The main strengths of this study were the large, randomly selected, nationwide cohort of non-pregnant, nulliparous women covering an age span of 40 years. The women included were aged 25–64 years, and older women were deliberately excluded to restrict the confounding effects of multiple illnesses. Subjects were identified and recruited by Statistics Sweden from the Total Population Register. All births are continuously recorded and updated every 6 weeks. A control question regarding ongoing pregnancy and previous births was also included in the questionnaire. A cohort restricted to nulliparous women is optimal for studying the natural history of pelvic floor dysfunction, without the interference of pregnancy- and birth-related confounding factors. All PFDs were self-reported using validated questions and reported according to current IUGA/ICS definitions [20]. The data presented were based on a subjective evaluation by the women themselves and were not confirmed objectively. However, the use of a questionnaire is considered to be the most feasible tool for gathering information about these sensitive issues [22]. The rate of childhood NE in the present cohort was 11.4%, similar to the 10.8% obtained by a meta-analysis of 15 studies on 22,140 7-year-old boys and girls, of whom 2392 had NE [2]. The question about childhood NE was answered by 98.6% of all women in this study.

Some limitations of this study need to be considered. First, the response rate increased consistently with age from 43% among the youngest to 63% in the oldest age group. A possible explanation for this difference in response rates is that older women may be more compliant and also more motivated to respond. Symptoms are more commonly encountered in older women, and they may have more spare time to respond. This may have tended to overestimate overall prevalence. Second, the validity of the self-reported information depends on the participants' willingness and ability to perceive, evaluate, and report correctly. Third, data about non-responders suggest a selection bias. Non-responders were younger, were more often immigrants or non-Swedish citizens, were less often married, were living in suburbs or commuting municipalities, had a lower income, and had a lower level of education compared with responders. A lower socioeconomic status (SES) among the non-responders is known to be associated with many other predictors of UI [2]. In addition, the women in this study were predominantly Caucasian, and the results should be interpreted with caution for diverse ethnic groups.

As in all questionnaire surveys involving past events, there was a risk of recall bias about CNE in this study. We observed that CNE was more prevalent in younger women compared to older women (Supplementary Material, Table S1). This is probably an effect of both forgetfulness with increasing age and a generational shift in attitudes toward the stigma of CNE. However, age did not affect the association between adult PFDs and CNE, as shown in Supplementary Material, Table S2. However, the response rate for the question was exceptionally high (99%), indicating that responders might be confident about their answers.

There are reasons to be skeptical about the rate of sPOP in women, both with and without CNE, in this study. It is known from a number of clinical studies that only 3 out of 607 nullipara women aged < 60 years had POPQ-stage ≥ 3 (0.5%), > 80% had stage 0–1, and 1 in 5 had a stage 2 prolapse [23–26]. Although it is common knowledge among gynecologists that the relationship between the anatomical stage of POP and sPOP is unpredictable, it has been shown that symptoms increase markedly once the leading edge reaches 1 cm proximal to the introitus, including most patients with POPQ-stage ≥ 2 [27]. In 199 women with POPQ-stage 0 (parity not specified), Tegerstedt et al. found that the “bulging” symptom was reported to occur “infrequently” in 6.0%, “sometimes” in 2.5%, and “often” in 0%, very similar to our results in almost 10,000 nulliparous women aged 25–64 years of age [16, 18, 21]. For women with POP-Q stage < 2, every third reported symptoms of “bulging,” of which 7% had POPQ-stage 0, i.e., were false positive [18]. Even if it is postulated that misdiagnosis with the “bulging” question, leading to false-positive responses, is not negligible, there is no obvious reason to reject the conclusion that the difference in prevalence between women with and without CNE reflects a real difference in the pelvic support.

In future epidemiological research, it would be interesting to define high- and low-risk subgroups of CNE for adult PFDs, e.g., those with early versus late spontaneous recovery of CNE, monosymptomatic versus non-monosymptomatic children with any concomitant lower urinary tract symptoms, more frequent bedwetters (once a week or more) compared to low-frequency bedwetters by age, and the interaction between CNE and childbirth.

## Conclusion

This study of PFDs in nulliparous women has shown that CNE was a significant predictor of a wide range of PFDs, indicating the possible existence of at least one common causal factor linking CNE to later PFDs in nullipara aged 25–64 years.

## Electronic supplementary material

ESM 1 (DOCX 21.5 kb)
