# Supplementary material for: Childhood nocturnal enuresis—a marker for pelvic floor disorders and urinary tract symptoms in women?
Source: Int Urogynecol J. 2020 May 30;32(2):359–65. doi: 10.1007/s00192-020-04345-x (PMC7838072; doi:10.1007/s00192-020-04345-x)
Supplement: Supplementary file 1 — (DOCX 21.5 kb) [file 192_2020_4345_MOESM1_ESM.docx]

**Supplementary material**

Table S1. Prevalence of childhood nocturnal enuresis according to age group

|  | 25-34 years  N = 3293 | 35-44 years  N = 2567 | 45-54 years  N = 1526 | 55-64 years  N = 1680 | *p* value * |
| --- | --- | --- | --- | --- | --- |
|  | n (%) | n (%) | n (%) | n (%) |  |
| CNE | 375 (11.4) | 287 (11.2) | 142 (9.3) | 122 (7.3) | < 0.0001 |
| Non-CNE | 2918 (88.6) | 2280 (88.8) | 1384 (90.7) | 1558 (92.7) |  |

Footnote: CNE denotes childhood nocturnal enuresis. *For comparison between groups the Mantel-Haenzel Chi Square test was used for ordered categorical variables.

Table S2. The association of childhood nocturnal enuresis on different pelvic floor disorders according to age group

| Outcome | 25-34 years  N = 3293 | 35-44 years  N = 2567 | 45-54 years  N = 1526 | 55-64 years  N = 1680 | Interaction  (age x enuresis) |
| --- | --- | --- | --- | --- | --- |
|  | OR (95% CI)* | OR (95% CI) | OR (95% CI) | OR (95% CI) | *p* value |
| UI | 2.67 (2.03; 3.52) | 2.59 (1.93; 3.47) | 2.47 (1.69; 3.61) | 2.10 (1.41; 3.13) | 0.33 |
| UI >10 years | 4.24 (2.60; 6.91) | 3.68 (2.24; 6.04) | 2.59 (1.03; 6.49) | 2.85 (1.34; 6.02) | 0.22 |
| Bothersome  UI | 3.41 (2.12; 5.47) | 4.59 (2.99; 7.06) | 1.63 (0.84; 3.15) | 2.02 (1.16; 3.50) | 0.0166 |
| Moderate and  severe UI**^§^** | 2.54 (1.63; 3.96) | 2.91 (1.96; 4.32) | 1.76 (1.07; 2.91) | 3.02 (1.96; 4.64) | 0.97 |
| SUI | 2.91 (2.01; 4.22) | 1.54 (0.98; 2.41) | 1.77 (1.05; 3.01) | 1.18 (0.62; 2.27) | 0.0255 |
| UUI | 3.34 (1.93; 5.77) | 2.64 (1.48; 4.70) | 1.70 (0.75; 3.88) | 1.70 (0.79; 3.64) | 0.10 |
| MUI | 2.02 (1.15; 3.55) | 5.02 (3.13; 8.07) | 2.17 (1.24; 3.78) | 2.06 (1.24; 3.43) | 0.34 |
| OAB | 2.37 (1.83; 3.06) | 2.69 (2.04; 3.55) | 2.53 (1.75; 3.64) | 1.69 (1.15; 2.48) | 0.25 |
| Bothersome OAB | 2.40 (1.62; 3.56) | 2.86 (1.91; 4.29) | 2.44 (1.49; 4.01) | 2.05 (1.28; 3.26) | 0.40 |
| sPOP | 1.73 (1.05; 2.84) | 2.57 (1.44; 4.58) | 1.34 (0.46; 3.90) | 1.37 (0.48; 3.92) | 0.77 |
| Bothersome sPOP | 3.52 (1.21; 10.22) | 0.64 (0.08; 4.94) | 2.32 (0.49; 11.08) | 1.33 (0.17; 10.63) | 0.43 |
| FI | 1.68 (1.22; 2.31) | 1.86 (1.32; 2.63) | 1.74 (1.13; 2.67) | 1.34 (0.83; 2.16) | 0.67 |
| Bothersome FI | 1.09 (0.54; 2.22) | 2.06 (1.04; 4.05) | 2.85 (1.41; 5.75) | 1.05 (0.41; 2.68) | 0.34 |
| Daytime micturition ≥8 | 1.24 (0.97; 1.59) | 1.38 (1.05; 1.80) | 1.25 (0.86; 1.83) | 1.73 (1.16; 2.58) | 0.19 |
| Nocturia ≥ 2 | 2.31 (1.48; 3.60) | 1.67 (1.11; 2.51) | 2.43 (1.52; 3.90) | 1.58 (1.01; 2.46) | 0.59 |
| One or more PFDs | 2.18 (1.72; 2.76) | 2.17 (1.67; 2.82) | 1.93 (1.35; 2.77) | 1.93 (1.32; 2.83) | 0.68 |

Footnote: OR denotes odds ratio, CI denotes confidence interval, UI denotes urinary incontinence, SUI denotes stress urinary incontinence, UUI denotes urge urinary incontinence, MUI denotes mixed urinary incontinence, OAB denotes overactive bladder, sPOP denotes symptoms of pelvic organ prolapse, FI denotes fecal incontinence, PFD denotes pelvic floor disorder (UI/sPOP/FI). **^§^**Moderate and severe UI is considered to be more severe forms of urinary incontinence according to Sandvik et al.^1^ *Adjusted for BMI (kg/m^2^).

Reference

1. Sandvik H, Hunskaar S, Seim A, Hermstad R, Vanvik A, Bratt H. Validation of a severity index in female urinary

incontinence and its implementation in an epidemiological survey. *J Epidemiol Community Health* 1993; 47: 497-9
